# Children still experience pain during hospital stay: a cross-sectional study from four countries in Europe

**DOI:** 10.1186/s12887-020-1937-1

**Published:** 2020-01-29

**Authors:** V. Vejzovic, J. Bozic, G. Panova, M. Babajic, A-C Bramhagen

**Affiliations:** 1 Faculty of Health and Society, Department of Care Science, Malmö University, SE-205 06 Malmö, Sweden; 2 School of nursing, Vinogradska, Zagreb, Croatia; 3 University Goce Delcev, Stip, Macedonia; 4 Clinic for Anaesthesiology and Rheumatology, University Clinical Center Tuzla, Tuzla, Bosnia and Herzegovina

**Keywords:** Children, Cross-sectional, Hospital stay, Pain, Pain assessment, Self-report

## Abstract

**Background:**

Little is known whether children experience pain during hospital stay from the child’s own perspective or not. The existing studies tend to be based on a small number of children and therefore have limitations concerning the generalisability of the results.

**Aim:**

The aim of this study was to describe children’s self-reported pain and experience concerning pain management during hospital stay.

**Methods:**

This study has a quantitative cross-sectional design with descriptive statistics as data analysis.

**Results:**

A total of 786 questionnaires, *Pain in Children in Hospital*, were distributed in four countries with the response rate of 75% which was almost equal between countries. Our result showed that 87% (503/579) children at hospital self-reported pain during the past 24 h. Nearly 63% of the children reported a pain score of > 5 the last 24 h. Most of children reported that they had received a question about pain from the hospital staff, and that the staff observed and assessed their pain. Totally 95% reported that they were satisfied with their pain relief during the last 24 h.

**Conclusion:**

Our study showed that when children were given the possibility to self-report pain, nearly 2/3 expressed that they had experienced pain during hospital stay. However, most of them reported satisfaction with pain management and their pain relief.

## Background

Children are a vulnerable group and their wellbeing should always be a priority [1]. Nevertheless, children have historically been exposed to many medical procedures and treatments without any analgesics since researchers claimed that small children did not feel any pain [2] despite the fact that the definition of pain is “a subjective, emotional, and unpleasant sensory experience” [3]. When Anand and Hickey [4] showed that a foetus had receptors for pain and that neonates could respond to pain in the same way as older children and adults, opportunities for further research in this field were obtained. Children who had been exposed to painful procedures without pain-relieving medication were found to have higher levels of stress hormones compared to those who had received medication [5]. Research has also shown that children who have experienced pain early in life may give a stronger response later in life when they for example receive a vaccination, as compared to children who have not experienced pain early in life and thus don’t have a similar “pain memory” [6]. Many children are inpatients at hospitals worldwide and several recently published studies have shown that children experience pain during hospital stay [e.g. [7–12]]. According to a study by Kozlowski et al. [8] children at surgical wards have reported more pain compared to children at medical wards (99 vs 65%). Furthermore, two studies have reported that children experience a fear of pain [13, 14].

World Health Organization [15] has established clinical guidelines for pain assessment and management but the knowledge and the attitudes of nurses regarding pain assessment and management has been identified as a potential barrier [16]. Results from a study by Manworren [16] where 247 pediatric nurses responded to a questionnaire about pain and pain management, showed that there were deficiencies concerning their knowledge about pain management. However, the nurses were aware of the children’s experience of pain (ibid). Hospitalised children often undergo not only painful procedures related to treatment, but they also experience pain related to their symptom which can result in distressing or negatively estimated memory [17]. The uncontrolled pain may cause lower patient and family satisfaction which may relate to longer hospital stays, frequent post discharge emergency room visits and early hospital readmissions [18].

Adequate pain management can reduce the anxiety of children and parents and increase compliance and collaboration [19] since even fear of pain is a problem [13, 14]. However, some studies suggest that gaps remain concerning nurse’s knowledge about pain in children and pain assessment [20] and that there is a lack of guidelines and specific protocols [21].

Unfortunately, despite the knowledge about pain and pain management, many children are still at risk for both poorer pain assessment and management [22] and the long-term effects of undertreating pain in children may result in psychosocial problems and recurrent pain syndromes [23].

Improving pain management requires a multifactorial approach concerning education, institutional support, attitude shifts and change leaders [24]. Pain assessment in younger children is often based on behavioral measures of pain observed by nurses or parents while self-assessment of pain is recommended in children older than 5 years [25]. Studies comparing patients’ self-assessment of pain and how nurses or other health professionals rate patients’ pain have shown that self-assessments and professional ratings often differ [26]. In 2015, Bramhagen and colleagues [27] developed an instrument according to PROM, patient-reported-outcome measures, in order to assess children’s (4–12 y) postoperative recovery, including pain, and their result showed that 23% (*n* = 55) of the children had answered the questionnaire themselves and 59% (*n* = 141) had participated “very much”. The instrument, Postoperative Recovery in Children, PRiC, proved to be useful for children when self-reporting how they felt after surgery.

Studies with children as inpatients are often performed as single-centre studies with small groups and therefore have limitations concerning generalisability [28]. Since results from previous studies show that too many children are still suffering from pain, larger studies with children involved are needed in order to minimize such limitations. An international collaboration with hospitals in multiple countries could make this possible. However, such a study would require a large sample size in different contexts and since such a study might be beset by a number of practical challenges, it is prudent to first undertake a feasibility trial. Thus, the aim of this study was to describe children’s self-reported pain and experience concerning pain management during hospital stay.

## Methods

This study has a quantitative cross-sectional design and was performed in four countries: Bosnia and Herzegovina (BH), Croatia (C), Macedonia (M), and Sweden (S). The present study was a feasibility study aimed to test a short questionnaire used for self-reported pain experience in children. If the children and/or the parents answered the questionnaire we presume that they understood the meaning of the questions and this was considered as a written consent to participate.

Ethics approval was collected in all four countries respectively. The study was approved by: The Swedish Ethical Review Authority in Lund (No; 2012/73), The Regional Ethical Review Board, Medical Sciences in Stip, Macedonien (No; 2015–225/02), University Clinical Center Tuzla; Sector for Scientific-Research and Professional Development (No; 2016/01), Clinical Hospital Center of Sestre milosrdnice Zagreb, Ethics Committee of KBSc of Sisters of Mercy, (No; 2016/06).

### Study population

All children (0–18 years of age) who were inpatients at different pediatric departments in community hospitals in Tuzla and Sarajevo – BH; Zagreb – C; Kocani and Stip – M; and Malmö – S, during a priori scheduled day were eligible for participation. The head nurse on each ward was responsible for excluding children who were too ill to participate or those who did not understand the official language. The head nurses were also asked to assign a nurse at the respective divisions responsible for distributing the questionnaire to the children and for collecting them. The children were asked to answer the questions by themselves and in case they needed help the parents were asked to assess/evaluate the pain from the child’s perspective. Prior to the data collection ethical issues were carefully considered and the national coordinators obtained ethical permission for all participating centers. All participants and their parents/caregivers were informed that participation was voluntary and by answering the questionnaire they gave their informed consent.

### Data collection

All children were inpatients at different pediatric wards in the respective countries. Since the study included several different contexts, one coordinator (JB) was responsible for all study activities in BH, C, M and S and main coordinators (ACB & VV) were responsible for the whole study. There was also a national coordinator (GP, MB, JB, ACB) in each country who had contact with the clinics and their staff during the data collection. The coordinators gave oral and written information about the study to the staff involved in data collection.

### Instrument

The questionnaire was inspired by a questionnaire used by Wadenstein et al. [29]. Wadenstein et al. included 1112 patient aged 6 w-95 years (mean 59 years) but only 4% were children. Therefore, the questions were revised, and formulated to suit a pediatric population (ACB), Pain in Children in Hospital, PiCH, and was then validated. In all, the instrument had seven questions whereas four questions had possible answers *Yes/No/Don’t’know*, two questions offered a scale *0–10 (no pain-worst pain*) and one question focus on satisfaction, *not at all - very good.* First, the content validity was tested by professionals, and secondly by a group of parents and their children to ensure that the questions addressed a pediatric population. The questionnaire was written in Swedish and tested in the Swedish context. Then it was translated into English in order to be translated into the native languages by the coordinators in the participating countries. Once translated to the local languages, the questionnaire was translated back to English; and finally, the original questionnaires and the back-translated questionnaires were compared (VV) to ensure that the meaning was not modified during the translation process [30].

### Data analysis

Descriptive statistics have been used to analyse the data. Frequency distribution in number, percent, and median was performed. A non-parametric Kruskal-Wallis test and Chi-square test were performed in order to compare the pain prevalence and pain assessment between groups (i.e. countries). A statistical significance of 0.05 was used throughout, and the software program SPSS statistics 22.0 (IBM Corp., Chicago, IL., USA) was used to perform all statistical analysis.

## Results

The planned distribution per country was 200 questionnaires. A total of 786 questionnaires were distributed. In all, 589 questionnaires were returned which gives a mean response rate of 75% for all four countries. The distribution of responses between countries were almost equal, Sweden (*n* = 138), Macedonia (*n* = 158), Bosnia and Herzegovina (*n* = 136) and Croatia (*n* = 156) and no significant differences were found. The number of subjects, response rate in each participating country and the distribution between boys and girls are shown in Table 1.

**Table 1:** overview of responses and gender distribution

	Croatia	Macedonia	Boznia-Herzegovinia	Sweden	Totally
Q/R^a^	200/156	200/158	200/136	182/138	588
M/F^b^	85/71	84/73	67/66	80/57	317/267

^a^ Delivered Questionnaire- returned questionnairs

^b^ Distribution between M (male) and F (female)

The children were asked to self-report or report with their parent’s help if they had pain (Yes/No), and to this 87% (503/579) reported pain during the past 24 h. There were significant differences (*p* = < 0.001) regarding prevalence of self-reported pain between the four countries, where Sweden had the lowest proportion of “Yes” with 68% (93/137) of the children having reported pain during the last 24 h. See Table 2.

**Table 2:** Overview of the result from all four countries

				Country		*p-*value
	Sweden	Macedonia	Croatia	Bosnia-Herzegovina	Totally	
Question	Yes/No	Yes/No	Yes/No	Yes/No	Yes/No	
1. Did you/your child had any pain during the last24 h during hospital stay?	93/44	150/7	148/0	111/25	503/76	< 0.001^a^
2. Did the staff asked you/your child about pain during the last 24 h?	79/18	156/0	153/2	127/4	516/24	< 0.001^a^
3. Did you/your child had the possibility to assess pain during the last 24 h with e.g. VAS,	22/75	60/96	124/30	18/114	225/315	< 0.001^a^
4. Did the staff observed your/your child’s pain in order to assess it?	51/45	156/0	151/1	129/3	488/49	< 0.001^a^
	0 = no pain			10 worst pain		
			median			
5. Assess your/your child’s pain by circling the number that best describes the pain when it was the most intense during the last 24 h?	6.00	5.00	7.00	5.00		< 0.001^b^
6. Assess your/your child’s pain by circling the number that best describes your/your child’s pain right now	2.00	3.00	3.00	3.00		0.001^b^
	*Not at all* % (n)	*a little %* (n)	*rather good* % (n)	*good* % (n)	*very good* % (n)	
7. How satisfied are you with your/your child’s pain relief during the last 24 hours?	2.7 (16)	6.8 (40)	24.6 (145)	28.5 (168)	29.2 (172)	

^a^Chi-square ^b^ Kruskal-Wallis

Children in Croatia had significantly higher scores for pain measured by a Visual Analog Scale, VAS (median 7.00) concerning the last 24 h (*p* < 0.001) as compared to the other three countries. Children in Macedonia and Bosnia and Herzegovina reported the lowest score for pain (median 5.00). There were also significant differences (*p* = 0.001) regarding “pain right now” where children in Sweden reported a median score at 2.00 and the other countries scored a median at 3.00. See Table 2.

Regarding question no 2, whether the child had received a question about pain from the hospital staff or not, 95% (516/589) had received the question but there were significant differences (*p* = < 0.001) between the countries. In Macedonia, all 156 children had received a question about pain, in Croatia nearly 99% (53/155), in Bosnia and Herzegovina nearly 97% (127/131) and in Sweden just above 81% (79/97). In all four countries, nearly 63% of the children reported a pain score of ≥5 during the last 24 h. Regarding pain right now, 14.9% reported no pain at all and 41% reported pain right now scored ≥5. Concerning the possibility to assess their own pain during the last 24 h, the result showed that there was significant difference (*p* = < 0.001) between countries where over 80% of the children in Croatia answered “Yes” (124/154) whereas more than 86% of the children in Bosnia and Herzegovina answered “No” (114/132). According to the children self-reporting (question no 4) there were significant (< 0.001) differences regarding if the staff observed and assessed the child’s pain or not. All children in Macedonia answered “Yes”, in Croatia over 99% answered “Yes” (151/152), in Bosnia and Herzegovina nearly 98% answered “Yes” (129/132) and in Sweden the distribution was almost equal between “Yes/No” (51/45). In summary, many children in Europe experience pain during hospital stay. Most of the children reported that they had received a question about pain from the hospital staff, and in C, M and BH, most of the children stated that the staff observed and assessed their pain, although only few children in BH (18/144) described that they were offered to assess their pain by a Visual Analog Scale, VAS. Totally 95% reported that they were satisfied with their pain relief during the last 24 h.

## Discussion

This study describes children’s self-reported pain, and experience concerning pain management during hospital stay. Poor patient recruitment is a well-known issue in clinical studies where children are patients and, the scientific literature has shown that the results of many studies are limited due to the small number of children involved in study populations. Our study showed that a short instrument with only seven questions and establishing an international network may be successful in clinical investigations in order to involve a higher number of participants. The response rate of 75% in this study can be seen as acceptable. Since all four countries were eager to perform this study and with good planning the recruitment of the participants was successful within the planned time.

The reason for conducting this study as a feasibility-study was the challenge in performing studies in different contexts and also to test the questionnaire in order to measure prevalence of pain in different counties. This study aimed to measure children’s self-reported experience of pain and it was important that both children and their parents understood the information given to them and the task instructions. To involve children in research concerning themselves is important according to UNICEF’s [1] recommendations so that the child’s rights will be respected. In this way, the child’s own voice can be heard, and nurses will be able to understand their needs and detect and minimize their pain during hospitalization. Our results showed an acceptable amount of returned questionnaires. However, we could not know whether or not the parents were involved in answering the questionnaire and if the instructions on how to report needed to be clearer. Our study has limitations, such as the lack of information concerning demographic data, time as inpatients and different diagnoses. Despite these limitations, this study provides important information concerning the feasibility to investigate pain prevalence and pain management in inpatient children from different countries. This study showed that we need to increase and strengthen information to all coordinators, maybe including 1 day of training, to make sure that all of them have similar concepts that will be used in the same way.

The value of preliminary work prior to organizing large-scale, globally randomised controlled trials [3] or intervention studies [31] have been shown earlier. UK National Institute for Health Research (NIHR) defines feasibility studies as studies used to estimate important parameters that are needed to design the main study, e.g., standard deviation of the outcome measure, response rates, willingness of clinicians to recruit participants and number of people eligible [32].

During this study, we did not experience difficulties with resources and the ability to manage the data collection; however, the data collection took longer time than expected.

Despite of different limitation, results from the present study showed that children are suffering from pain during hospitalisation today although there is a rich amount of knowledge about children and pain. Regardless of rapid development of pediatric pain management in pediatric care during the last three decades, there are still studies revealing that pediatric patients might not always receive optimal pain management [7, 10, 28]. Most of the children in the present study reported that the staff observed and assessed their pain, but it is unclear if the staff used any pain scale when they assessed pain or if they evaluated any given pain medication. However, it would be valuable to understand if, and if so how the staff assessed and documented their observations. Despite the knowledge about pain in children there is a gap between available research evidence and the best available evidence used in regular clinical practice. This phenomenon, known as the knowledge-to-action gap, has been described in many areas of medicine and health [33]. Our study confirmed that this phenomenon still exists and that it is important to understand why, which is why more research is needed. The results of this study show that pain in children as inpatient exists in more than 80% of the cases. Our results also exhibit differences between countries; however we believe that these differences are difficult to interpret since there are possible influencing factors that are unknown. Building on the results of the current study, it will be important to conduct a large-scale study with an extended number of questions where both parents and staff respond to the questions about pain and pain assessment.

Our result have generated new questions and therefore, we are starting a new project in this area including five countries with a complementary study aiming to investigate nurses’ attitudes toward children and information about pain management and care practices. Since nurses usually are responsible for children’s pain during hospital stay and during different medical procedures, her/his attitude are of greatest interest in how the pain management are being handled and to what extend it is successful*.*

## Conclusions

Our study showed that when children were given the possibility to self-report pain, nearly 2/3 expressed that they had experienced pain during hospital stay. However, most of them reported satisfaction with pain management and their pain relief. In conclusion, this study demonstrated that self-reported pain in inpatient children is vast in S, M, C and BH. This work highlights the importance of future studies on pain assessments in different pediatric areas.

## Data Availability

All the data is presented in the main manuscript. The datasets used and/or analysed during the current study are available from the corresponding author on reasonable request.

## Abbreviations

BH: Bosnia and Herzegovina
C: Croatia
M: Macedonia
PROM: Patient-reported-outcome measures
S: Sweden
VAS: Visual Analog Scale
